# Vestibular bone thickness of the mandible in relation to the mandibular canal—a retrospective CBCT-based study

**DOI:** 10.1186/s40729-019-0189-z

**Published:** 2019-11-15

**Authors:** Silvio Valdec, Jan M. Borm, Stephanie Casparis, Georg Damerau, Michael Locher, Bernd Stadlinger

**Affiliations:** Clinic of Cranio-Maxillofacial and Oral Surgery, Centre for Dental Medicine, University of Zurich, Plattenstrasse 11, 8032 Zurich, Switzerland

**Keywords:** CBCT, Inferior alveolar nerve, Mental foramen, Bone transplantation, Dental implant

## Abstract

**Background:**

The aim of this study was to assess vestibular bone thickness of the mandible in relation to the mandibular canal and position of the mental foramen in relation to the neighbouring teeth. Measurements were performed on radiographic cone-beam computed tomography (CBCT) images.

**Methods:**

This retrospective study analysed 314 CBCTs, having been taken at the Clinic of Cranio-Maxillofacial and Oral Surgery, University of Zurich, Switzerland.

**Results:**

CBCTs from 168 female and 146 male patients (median age 40.2 years) were analysed. Median bone thickness lateral to the nerve canal to the buccal mandibular cortical plate was ~ 4 mm immediately posterior to the mental foramen, increased to ≤ 6 mm over the next 30 mm, then decreased to ~ 3 mm at the level of the mandibular foramen. In two thirds of cases, both mental foramina were located near the second premolar (66.2% right, 67.7% left). Bone thickness and the position of the mental foramen showed marked intra- and interindividual variance.

**Conclusions:**

A preoperative CBCT is recommended for detailed planning of surgical interventions that may reach the mandibular canal (e.g. wisdom teeth removal, root resection, implant placement, bone block harvesting).

## Background

When performing any kind of surgical procedure, a surgeon needs to be familiar with the possible variations in the anatomical configurations of both the mandibular canal and inferior alveolar nerve (IAN) [1–3]. This is particularly the case when performing root resections, removing wisdom teeth or harvesting autologous bone grafts.

Different techniques are described for reconstruction of missing areas of bone before or during implant insertion. Autogenous bone, i.e. a block graft, is often used. The block can be obtained intra- or extraorally [4, 5]. Extraoral bone harvesting, e.g. from the hip area (anterior superior iliac crest), requires general anaesthesia, causes higher costs and takes more time. Such a procedure is associated with a hospital stay (often of several days), temporary walking difficulties and an additional scar in the area of bone removal and sometimes with sensory disturbances in the thigh. Intraoral bone harvesting, which can be performed under local anaesthesia, may be suitable for obtaining a graft for localized bone defects [6–9]. The most common harvesting site is the vestibular retromolar area of the mandible in the area of the external oblique line. Anatomically, the IAN runs significantly close to the vestibular bone surface in the area of the ascending mandibular ramus. This nerve may be exposed during bone harvesting if the external oblique line is less pronounced or the bone block preparation extends below the course of the nerve. The nerve may also be exposed if the distal vertical incision is located in the area of the ascending mandibular ramus, because the IAN runs close to the buccal cortical plate before entering the mandibular body in a lingual direction. Other intraoral harvesting sites for bone blocks are the area of the premolars or the chin [10]. Various methods can be used to remove the graft from the donor region; piezosurgery and use of a trephine drill or a Lindemann drill are particularly worthy of mention. The benefits of such a procedure always have to be weighed up against the risks. Various complications are described in the literature, such as damage to teeth, sensory disturbances in the skin or mucous membrane, excessive keloid formation, postoperative complaints (restricted mouth opening, secondary haemorrhage, swelling and pain) or aesthetic problems (altered profile in the area of the donor region or soft tissue recession) [11–13]. Possible damage to the IAN during block harvesting and other procedures that may reach the mandibular canal is a feared complication and is the focus of this paper.

The mandibular canal is a bilateral, intraosseous opening through which the IAN runs from the mandibular foramen to the mental foramen. The nerve innervates the teeth, the mucous membranes in the area of the mental foramen and the skin around the chin [14, 15]. Anatomical variations of the mandibular canal, such as bifid canals and an anterior loop of the mental nerve, are common [16, 17] and have to be considered when planning surgical interventions (e.g. implantations, osteotomies) in the area of the mandible [14, 15, 18, 19]. The literature contains reports of sensory disturbances within the first 2 weeks after surgical interventions involving block harvests in up to 37% of patients, 10–15% of whom continue to have persisting complaints after 15 months [19, 20]. Other authors report milder complications after bone harvesting in the retromolar area of the mandible compared to the chin area [5, 12, 21–23].

The objective of this study was to evaluate the bone thickness between the lateral wall of the mandibular canal and the buccal side of the vestibular cortical plate of the mandible, i.e. the horizontal position of the mandibular canal, and to determine the position of the mental foramen in relation to the neighbouring teeth. The position of the mandibular canal is of particular interest in bone block harvesting in the posterior mandible, implant placement and root resection on mandibular premolars or molars. Preoperative three-dimensional diagnostics by CBCT is an essential planning tool to evaluate the donor region and was analysed in this study.

## Methods

### Study design and data collection

Three hundred fourteen cone-beam computed tomograms (CBCTs) from 168 (53.5%) females and 146 (46.5%) males from the database of the Department of Cranio-Maxillofacial and Oral Surgery, Center of Dental Medicine, University of Zurich, Switzerland, from the years 2008 to 2013 were analysed. Patients were divided into 4 age groups: group 1 = 0–20 years, group 2 = 21–40 years, group 3 = 41–60 years and group 4 = 61 years and older.

Inclusion criterion was the presentation of the entire mandible. All images were taken with the 3D eXam CBCT (KaVo Dental GmbH, Biberach, Germany) and evaluated with the KaVo eXamVision Software (Version 1.9.3.13, KaVo Dental GmbH). The X-ray parameters were 120 kV at 3–8 mA (pulsed); image resolution was between 0.25- and 0.4-mm voxel side length. The data was processed in DICOM format on a PC (IntelR CoreTM i5-2500 CPU @ 3.3 GHz, 4 GB RAM, Windows 7 operating system) and evaluated on a diagnostic monitor.

Clinical indication for the CBCTs included the assessment of wisdom teeth, pre-implant assessment, diagnosis of intraosseous pathologies and exclusion of fractures. Exclusion criteria for this study were an impaired continuity of the IAN or an ambiguous course of the nerve and an irregular bone volume caused by pathologies in the area of the planned measurements.

### Measurement procedures

**Fig. 1:**
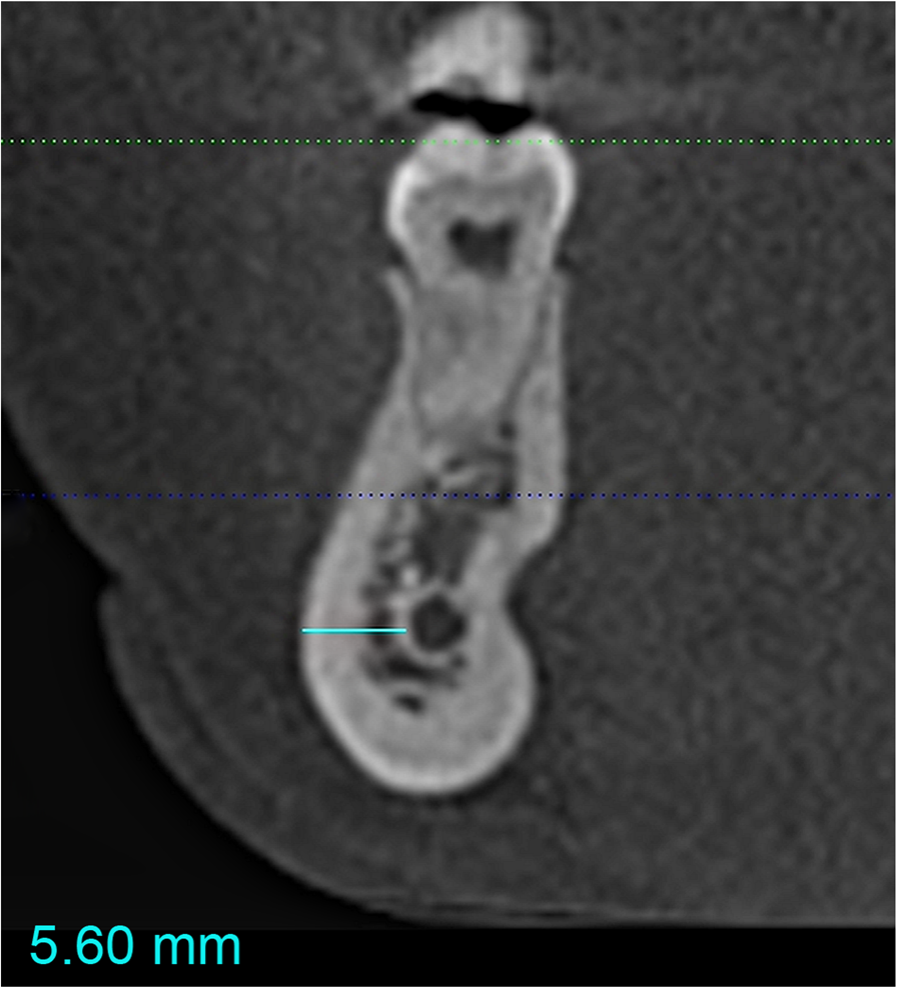
Measurement of mandibular bone thickness, defined as the distance between the lateral wall of the mandibular canal and the lateral mandibular compact bone (solid turquoise line)

The CBCTs were evaluated under standardized conditions by an investigator, being familiar with the software. First, the complete course of the IAN was evaluated in every CBCT, then bone thickness lateral to the nerve canal (spongious bone + cortical plate) was measured along the full length of the IAN. Measurements were performed at 2-mm intervals on both sides of the mandible in a sagittal direction along the whole course of the nerve, starting 2-mm distal to the posterior edge of the mental foramen on each side and ending at the mandibular foramen. At each measuring point, the lowest vestibular bone thickness (bt) was recorded as the shortest possible horizontal distance between the lateral wall of the mandibular canal and the buccal mandibular compact bone (see Fig. 1).

**Fig. 2:**
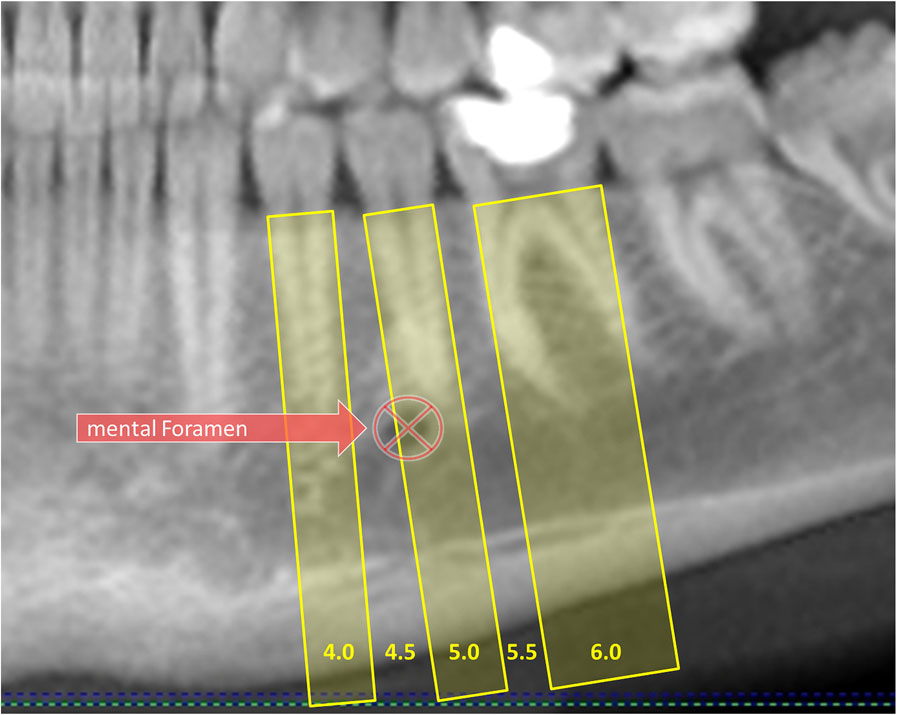
Definition of the position of the mental foramen

In addition, the position of the mental foramen was determined relative to the roots of the neighbouring teeth. This was assessed by defining regions of interest in the area of the first premolar, second premolar and first molar by extending the respective mesial and distal points of the cement-enamel junction caudally along the tooth axis. The position of the midpoint of the mental foramen was then determined in relation to these regions to determine its position relative to the roots (see Fig. 2).

### Data and statistical analysis

SPSS 20.0 software (IBM) was used for statistical analysis. The medians, mean values, standard deviation and confidence intervals of all measurements (distance from the mandibular canal to the vestibular compact bone of the mandible and the position of the mental foramen) were calculated. Wilcoxon rank-sum test was used for comparing the means of all bone thickness measurements between men and women, the different age groups and the right and left sides of the mandible.

## Results

The median age of the patients was 40.2 years (range 12.6–84.4 years). Patients were distributed almost evenly across the age groups (see Table 1).

**Table 1:** Number of men and women in each age group (group 1, 0–20 years old; group 2, 21–40 years old; group 3, 41–60 years old; group 4, 61 and older)

		Age group				Total
		1	2	3	4	
Sex	Male	41	36	39	30	146
	Female	44	51	35	38	168
Total		85	87	74	68	314

**Fig. 3:**
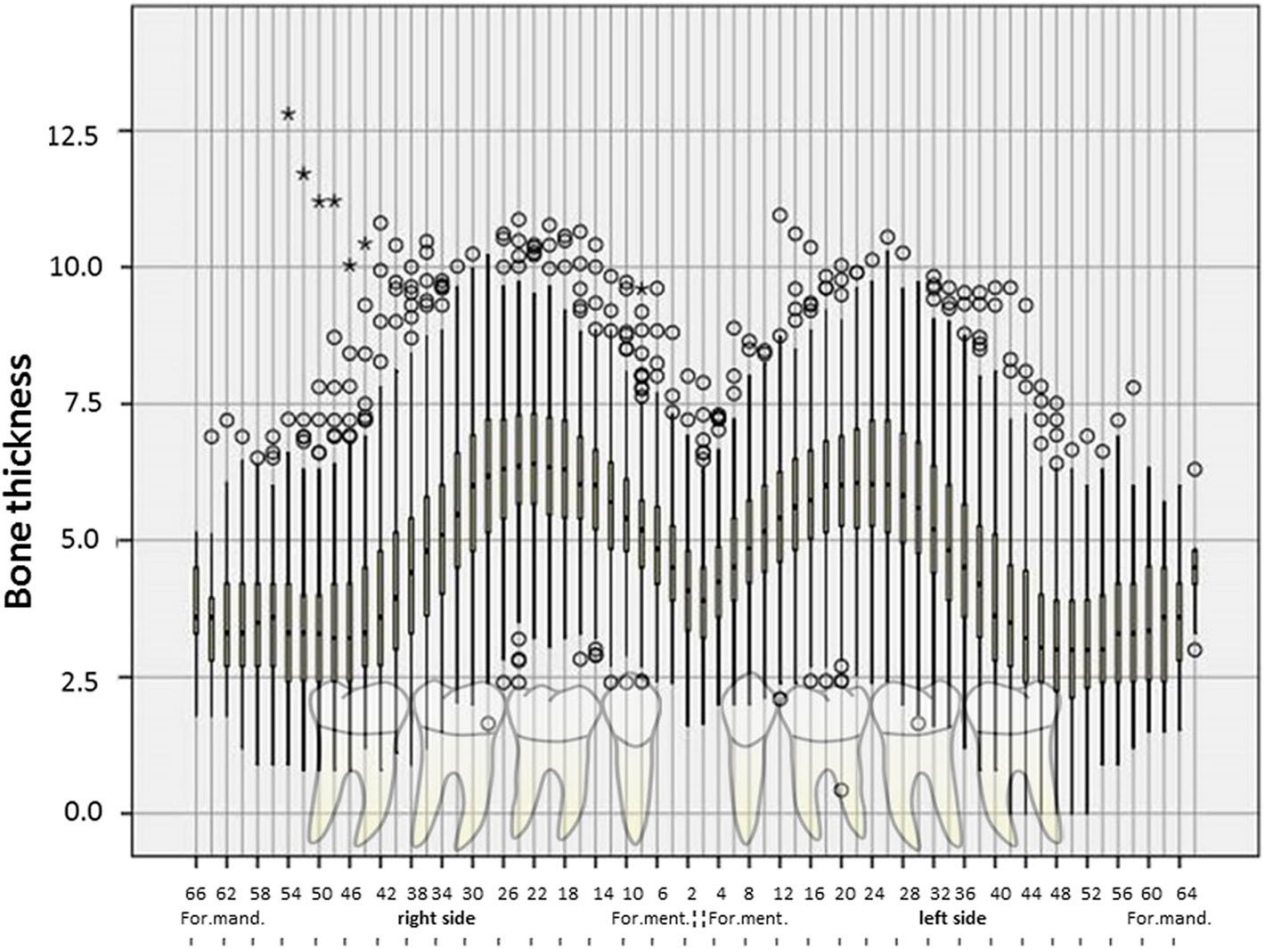
Left (l) and right (r) mandibular bone thickness in all patients

Figure 3 clearly shows the median vestibular bone thicknesses (bt) at 2-mm intervals throughout the anterior to posterior course of the canal on both the right (bt2 r to bt66 r) and left (bt2 l to bt66 l) side of the mandible. The maximum distance between the mental and mandibular foramina was 6.6 cm. As shown in Fig. 3, the vestibular bone thickness on both sides is approximately 4 mm immediately behind the mental foramen, increases to 6 mm in further distal course and is approximately 3 mm towards the posterior at the mandibular foramen. Some individual measurements differed significantly from the median values. For example, immediately posterior to the right mental foramen, the bone thickness ranged from 1.6 to 8 mm.

**Fig. 4:**
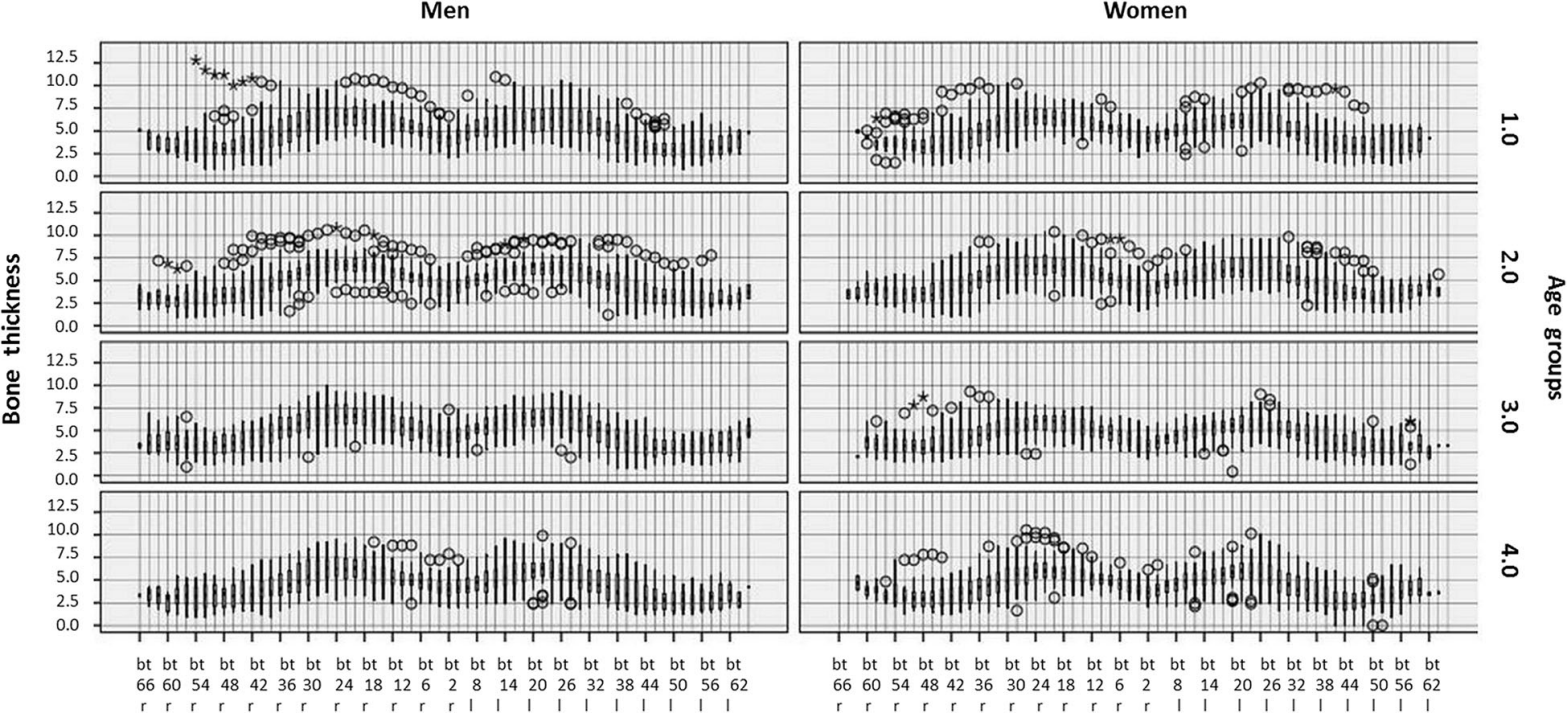
Left (l) and right (r) mandibular bone thickness (bt) in the four age groups (group 1, 0–20 years old; group 2, 21–40 years old; group 3, 41–60 years old; group 4, 61 and older) in men (0) and women [1]

We found some highly significant differences (*p* < 0.001) in bone thickness between the right and left side of the mandible in both men and women (see Fig. 4). Bone thickness also varied significantly between both men and women (*p* < 0.05), particularly in the first 30 mm posterior to the mental foramen (bt14 r to bt26 r and bt12 l to bt 28 l).

Bone thickness did not differ between the age groups among men. However, among women, significant differences were found in the first 40 mm posterior to the mental foramen on both sides of the mandible when comparing age groups 2 and 3, 3 and 4, and 2 and 4 (*p* < 0.01 in all cases).

**Fig. 5:**
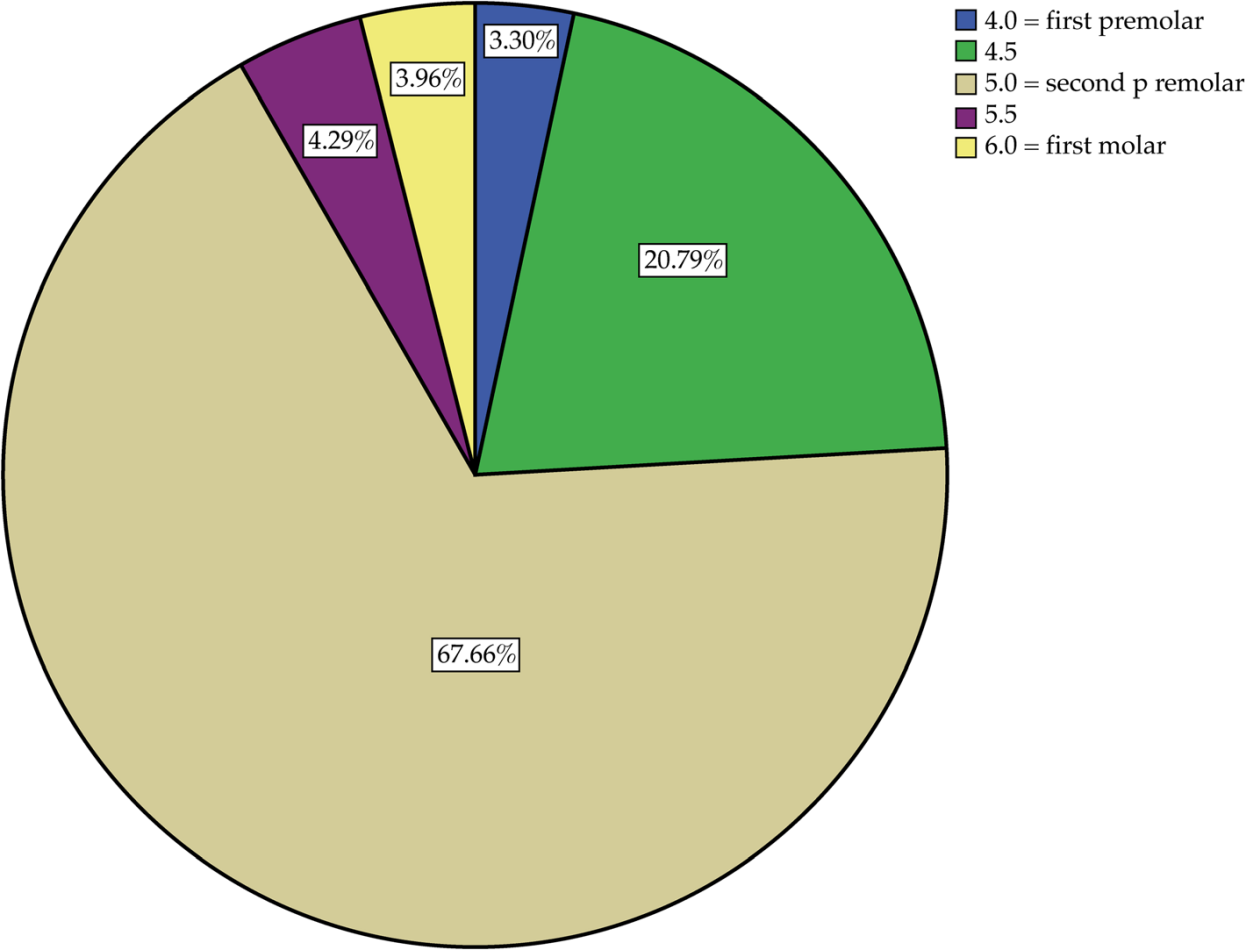
Position of the left mental foramen

**Fig. 6:**
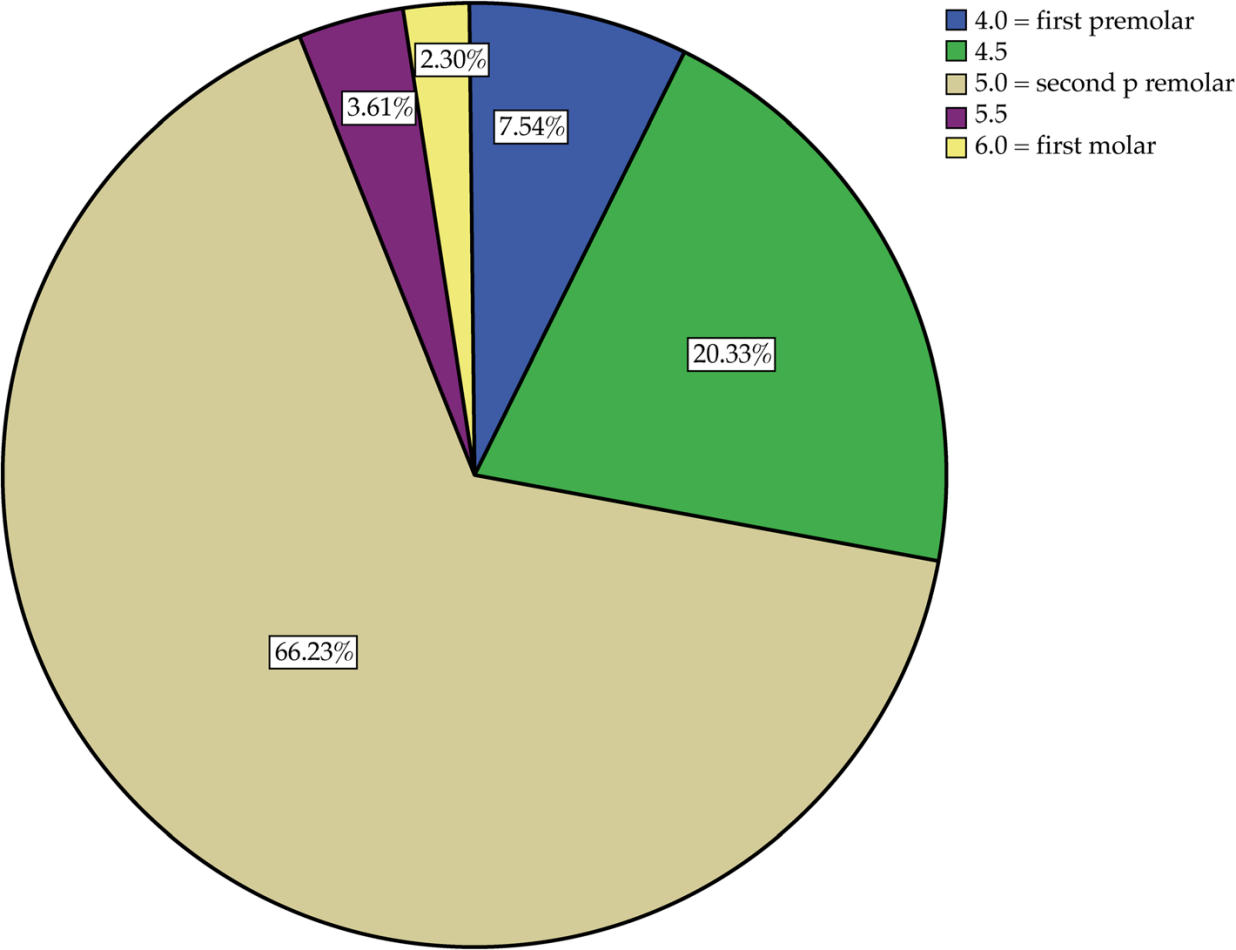
Position of the right mental foramen

In all CBCTs, the mental foramen was visible on the right and left side of the mandible and in two thirds of the cases (66.23% right, 67.66% left) was located near the second premolar. The location was not completely symmetrical: the foramen was more often mesial to the second premolar on the right side (27.87%) than on the left side (24.09%) and significantly more often distal to the second premolar on the left side (8.25%) than on the right side (5.91%) (*p* < 0.001) (see Figs. 5 and 6). Further analysis showed that gender did not affect the location of the foramen.

## Discussion

The IAN is an important anatomical structure whose course affects the preoperative planning of a bone graft or implant insertion in the mandible. Knowledge on the bone thickness between the lateral wall of the mandibular canal and the lateral mandibular compact bone as well as of the position of the mental foramen facilitates decision-making [24]. Furthermore, for many other surgical procedures, the overall dimension of the mandible is crucial. This is the case in the removal of wisdom teeth, in the application of buccal mini screws for orthodontic anchorage and of course in orthognathic surgery [25–27]. Therefore, this study used CBCTs to assess the intraosseous course of the IAN, the bone thickness between the lateral wall of the mandibular canal and the buccal border of the mandibular cortical plate and the position of the mental foramen in relation to the neighbouring teeth. The findings were evaluated for men and women and different age groups, and both sides of the mandible were compared.

Analysing bone thickness, our CBCT-based study measured the distance between the mandibular canal and the buccal bone surface of the mandible. The measurement of other distances like, e.g. the length between the mandibular canal and the lingual, apical and cranial mandibular bone surface, was performed in various cadaver studies [28–30]. These studies show a 2.1–5.8-mm distance between the lingual bone surface to the mandibular canal, 8.2–21.3 mm for the upper border (alveolar crest) to the mandibular canal and 6.2–11.8 mm for the lower border to the mandibular canal as summarized by von Arx and Lozanoff [31]. Chrcanovic et al. performed preoperative measurements of the distances between the buccal and lingual bone surface to the mandibular canal using CBCTs, underlining the clinical relevance of the distance between the buccal wall and the mandibular canal due to an increased risk of nerve damage at short distances [32, 33].

In a study comparing measurements between cadavers and CT images, the distance between the upper edge of the mandibular canal and the alveolar ridge showed possible over- and underestimations. The quantification showed a possible overestimation of up to 1.05 mm and a possible underestimation of up to 1.36 mm [34]. This discrepancy is of relevance in preoperative planning. Intraoperatively, a risk of bone block harvesting is the damage to the IAN, depending on the depth or angulation of the osteotomy. Hanser and Dolliveux describe further complications like bone overheating and damage due to chisel placement. Such complications can be avoided, knowing about the patient’s individual anatomy with regard to the mandibular canal and the osteotomy [35].

In the present study, the median bone thickness between the mandibular canal and the buccal surface of the mandibular cortical plate was approximately 4 mm immediately posterior to the mental foramen. This distance increased up to 6 mm in the first 30 mm posterior to the mental foramen and decreased to about 3 mm at the most posterior measurement at the level of the mandibular foramen. Large inter-individual differences in bone thickness were found. The findings of this study indicate that the vestibular bone thickness, i.e. the vestibular distance to the mandibular canal, is generally greatest 30 mm posterior to the mental foramen.

Significant differences in bone thickness between the right and left mandibular side support the known asymmetry of the two halves of the face [36]. The significant differences between men and women, mainly in the region of the first 30 mm posterior to the mental foramen, indicate that the mandible is wider in the area of the mental foramen in men. In contrast to women, males did not show significant age-related differences in bone thickness within this study.

In two thirds of the cases, the mental foramen was located in the region of the second premolars (66.2% right, 67.7% left) (see Figs. 5 and 6). Interestingly, the location was not completely symmetrical: the mental foramen was distal to the second premolar significantly more often on the left side than on the right side and mesial to the second premolar more often on the right side than on the left side. This finding is in agreement with those of Phillips et al. [37] and Pyun et al. [38]. In the present study, gender showed no effect on the position of the mental foramen; however, there was a trend towards an effect for age.

## Conclusions

The results of this study support the relevance of a preoperative CBCT to allow detailed planning of a surgical intervention that may potentially touch the area of the mandibular canal. This applies to surgical procedures like wisdom tooth removal, root resection, implant placement and bone block harvesting. A CBCT allows the exact determination of the horizontal bone thickness vestibular to the IAN, and this may avoid potential damage to the nerve.

## Data Availability

The original datasets supporting the findings are available.

## Abbreviations

bt: Bone thickness
CBCT: Cone-beam computed tomography
DICOM: Digital Imaging and Communications in Medicine
IAN: Inferior alveolar nerve
